# Plant peptides – taking them to the next level

**DOI:** 10.1093/jxb/erw309

**Published:** 2016-08-13

**Authors:** Barbara De Coninck, Ive De Smet

**Affiliations:** 1 Centre of Microbial and Plant Genetics, KU Leuven, Leuven, Belgium; Department of Plant Systems Biology, VIB, Ghent, Belgium; 2 Department of Plant Systems Biology, VIB, Ghent, Belgium; Department of Plant Biotechnology and Bioinformatics, Ghent University, Ghent, Belgium

**Keywords:** Cell-to-cell communication, cysteine-rich peptides (CRPs), plant hormones, post-translationally modified (PTM) peptides, secreted peptides, signalling pathways, small signalling peptides

**Plant growth, development, reproduction and environmental stress responses are tightly regulated by a complex network of signalling pathways. Plant hormones – including salicylic acid, ethylene, jasmonic acid, auxins, gibberellins, cytokinins, abscisic acid and brassinosteroids – have long been considered the major signalling molecules during those processes. However, the discovery that many different (secreted) peptides are involved in signalling has stimulated intensive research, and this special issue reflects the latest developments in this dynamic field.**

Many different secreted peptides can fulfil a role as regulators of signalling events and cell-to-cell communication in plants (Murphy *et al.*, 2012; Albert, 2013; Czyzewicz *et al.*, 2013; Matsubayashi, 2014). Most small signalling peptides are derived from larger inactive precursor proteins with an N-terminal signal sequence directing the protein to the secretory pathway. Precursor proteins can also contain prodomains, requiring additional processing to obtain the biologically active mature peptides (Tavormina *et al.*, 2015). However, little is known at present about the proteases involved in the maturation process.

Small signalling peptides are mainly classified into two groups, the cysteine-rich peptides (CRPs) and post-translationally modified (PTM) peptides. The latter peptides typically consist of a maximum of 20 amino acids and are altered by modifications such as tyrosine sulfation, proline hydroxylation and hydroxyproline glycosylation. CRPs contain from 2 to 16 Cys residues and each CRP class has a characteristic number and linear arrangement of these amino acids. While it was previously thought that CRPs mainly function as antimicrobial compounds during plant–microbe interactions [reviewed in Van Der Weerden *et al.* (2013) and Tavormina *et al.* (2015)], they have also been reported to have essential roles in stomatal patterning and density, symbiosis and a wide range of reproductive processes such as pollen tube germination, guidance and burst, gamete activation, and seed development (Hara *et al.*, 2007; Sugano *et al.*, 2010; Maróti *et al.*, 2015; Bircheneder and Dresselhaus, 2016). In line with a role in reproduction, CRPs are overrepresented in both female and male gametophytes, in contrast to PTM peptides, which occur predominantly in vegetative tissues.

Bircheneder and Dresselhaus (2016) provide strong arguments for the hypothesis that several CRPs evolved from antimicrobial peptides (AMPs) towards signalling peptides during reproduction, with a highly conserved mode of action in both processes. Compared to the exponential growth in knowledge on small signalling peptides, research on the mode of action of plant AMPs is limited to a few families, including the defensins, which are characterized by an α-helix and a triple-stranded β-sheet stabilized by four disulfide bridges (Vriens *et al.*, 2014; see also a brief description in Bircheneder and Dresselhaus, 2016), and cyclotides, which are cyclic peptides with a head-to-tail backbone and three disulfide bridges. The remarkable structure of cyclotides provides them with exceptional properties of stability, which are being exploited for peptide-based applications in the pharmaceutical and agricultural industries (Weidmann and Craik, 2016).

This special issue of *Journal of Experimental Botany* builds on the one from last year (see Simon and Dresselhaus, 2015), which provided a broad overview of the roles and downstream effects of different plant peptide classes. Here, we mainly focus on the small signalling peptides, including the CLAVATA3/EMBRYO SURROUNDING REGION (CLE), C-TERMINALLY ENCODED PEPTIDE (CEP), RAPID ALKALINIZATION FACTOR (RALF) and ROOT GROWTH FACTOR/CLE-LIKE/GOLVEN (RGF/CLEL/GLV) peptides, providing more information on their biological functions, downstream effects, processing and perception as increasing numbers of research projects have rapidly expanded our knowledge (Box 1).

**Box 1. Overview of peptide signalling and activity**

The simplified schematic indicates some of the key points at the level of the (small signalling) peptide, receptor complex and downstream response. Two adjacent cells (light grey) separated by a cell wall (dark grey) are shown.

**Figure:**
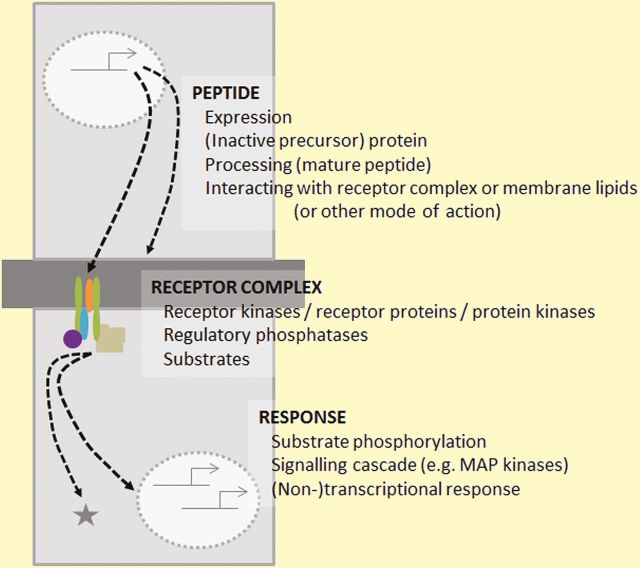

## Involvement in the whole plant life cycle

The CLE peptides are probably the best-studied family of PTM peptides. Mature CLE peptides contain 12 to 13 amino acids and have been found in various plant species. Functional roles in maintenance of root, shoot and floral meristems, lateral root emergence and vascular development have been reported for about half of the 32 CLE peptides identified in Arabidopsis, but CLE signalling pathways are also involved in plant–environment interactions, including symbiosis and responses to abiotic stress (Mitchum *et al.*, 2008; Wang and Fiers, 2010; Kiyohara and Sawa, 2012; Miyawaki *et al.*, 2013; Qiang *et al.*, 2013; Yamaguchi *et al.*, 2016).

Intriguingly, CLE-like peptides that alter plant morphology have also been identified in nematodes (Mitchum *et al.*, 2008; Kiyohara and Sawa, 2012; Miyawaki *et al.*, 2013; Yamaguchi *et al.*, 2016). Furthermore, very recently, RALF-like peptides have been identified in two fungal pathogens of poplar (Thynne *et al.*, 2016), and the root-infecting fungus *Fusarium oxysporum* is now known to use a functional RALF homologue (Masachis *et al.*, 2016). It seems that the acquisition of signalling peptides by plant-interacting organisms, enabling the host-cell machinery to be hijacked, is a more general concept than initially thought.

RALF peptides are CRPs of about 5kDa that affect cell and organ growth via the elicitation of Ca^2+^ responses, activation of MAPK signalling and pH modulation (Murphy and De Smet, 2014). Although RALF peptides have previously been linked to lateral root development (Bergonci *et al.*, 2014), Murphy *et al.* (2016) now reveal, for the first time, the importance of a RALF-LIKE peptide (RALFL34) in lateral root initiation. Interestingly, experiments focusing on transcriptional regulation – something that has been investigated only in a very limited way for small signalling peptides – revealed the involvement of APETALA2/ETHYLENE RESPONSIVE FACTOR (AP2/ERF) transcription factors, downstream of auxin. In addition, it appears that *RALFL34* expression is an earlier marker for lateral root initiation than *GATA23*, and could play a key role in interpreting a shoot-derived signal that is involved in positioning lateral roots along the primary root axis.

Another family of signalling peptides with a role in lateral root development, in addition to their role in primary root and shoot growth and root nodule development, are CEPs, which are PTM peptides consisting of 15 amino acids (Ohyama *et al.*, 2008; Delay *et al.*, 2013; Imin *et al.*, 2013; Mohd-Radzman *et al.*, 2015). Roberts *et al.* (2016) have identified – from targeted transcriptome data – the auxin-repressed *CEP5* as a negative regulator of lateral root initiation (see also the Insight article on this by Taleski *et al.*, 2016). Interestingly, *CEP5* is expressed at the phloem pole, but still seems to have a strong impact on lateral root initiation and development. Using selected reaction monitoring, the authors were able to demonstrate the presence, *in planta*, of a 15-amino-acid CEP5 peptide with three Hyp residues. In future, such approaches will probably be increasingly used to supplement more global LC-MS-based peptidomics analyses.

A more recently identified family of PTM peptides are the RGF/CLEL/GLV peptides, with previously assigned roles in root gravitropism, maintenance of the root apical meristem, and root hair, lateral root and shoot development (Matsuzaki *et al.*, 2010; Whitford *et al.*, 2012; Meng *et al.*, 2012; Fernandez *et al.*, 2013, 2015). These peptides are also now known to promote cell elongation in the growing hypocotyl (Ghorbani *et al.*, 2016). Mature RGF/CLEL/GLV peptides are derived from a preproprotein with an N-terminal signal sequence, a conserved RGF/CLEL/GLV C-terminal domain and a variable prodomain. The latter carries sites that may be targeted by subtilases, proteases that are responsible for maturation of other signalling peptides, including PHYTOSULFOKINE4 and RALF23 (Srivastava *et al.*, 2008, 2009). The *GLV1* overexpression agravitropic curly root phenotype allowed Ghorbani *et al.* (2016) to use a suppressor screen on knock-out lines of subtilase genes to pinpoint the specific proteins responsible for maturation of the GLV1 proprotein. As such, two related subtilase (SBT6) genes were identified and their role in cleavage of the GLV1 proprotein confirmed with an *in vitro* protease assay. The authors also provide clear indications that SBT6 is under the control of the SERPIN1 protease. Hence, production of the active GLV1 peptide depends on the activity of both subtilase and its inhibitor, SERPIN1.

It can be concluded that functional roles of signalling peptides are increasingly understood in roots, a model organ that lends itself to developmental study because of its simple cellular organization and easy growth in non-soil media, which facilitates phenotypic analyses. Although an increasing number of reports indicate that signalling peptides are involved in every aspect of a plant’s life cycle, functional studies are often hampered by the lack of suitable knock-out lines. These would greatly benefit from current genome-editing technologies such as the CRISPR/Cas9 system, allowing specific mutations in the critical coding regions of the peptides (Yamaguchi *et al.*, 2016).

## New developments in peptide perception

Perception of secreted signalling peptides is complex and involves multiple plasma membrane-localized receptors, generally identified as leucine-rich repeat (LRR) receptor-like kinases (LRR-RLKs). However, RLKs without an LRR-domain or receptors lacking a kinase domain exist, indicating that multimeric complexes need to or can be formed. Moreover, signalling peptides can often be recognized by more than one RLK, or vice versa. One of the most intensively studied ligand–receptor pairs is the interaction between CLAVATA 1 (CLV1)-type receptors and CLE peptides, such as CLV3, controlling stem cell fate in apical meristems [this is comprehensively reviewed by Hazak and Hardtke (2016) and Yamaguchi *et al.* (2016)].

CLV3 is perceived by multiple complexes including the homomer CLV1-CLV1, the heteromer CLV2-CRN (CORYNE) and the multimer CLV1-CLV2-CRN receptors (Somssich *et al.*, 2015). While CLV1 is a genuine LRR-RLK, CLV2 lacks the kinase domain and CRN is devoid of an extracellular receptor domain, indicating that CLV2 and CRN could interact to form a functional receptor complex. However, CRN has been shown to be a pseudokinase and conflicting results have been reported on the ability of CLV2 to bind CLV3 (Nimchuk *et al.*, 2011; Shinohara and Matsubayashi, 2015). The study of Somssich *et al.* (2016) provides evidence that CRN is actively involved in CLV3 peptide signal transduction and that the mode of action of CRN differs between shoot and root meristems. The authors propose an interesting model of the different receptor complexes involved in CLE peptide signalling. In shoot meristems, the kinase domain of CRN (together with CLV1 and CLV2) is essential for its function in CLV3 signalling, while in root meristems, the CRN kinase domain is not required and the CLV2-CRN complex functions independently of CLV1, probably with another, as yet unknown, RLK. It has been suggested that other LRR-RLKs may also play important roles in CLV3 signalling, including RECEPTOR-LIKE PROTEIN KINASE 2 (RPK2) and BARELY ANY MERISTEM 1 and 2 (BAM1 and 2) (Shimizu *et al.*, 2010, 2015; Kinoshita *et al.*, 2010; Shinohara and Matsubayashi, 2015).

Furthermore, in Arabidopsis, the RLK CRINKLY4 (CR4) has been suggested to play a role in CLE signalling, more specifically involving CLE40, making the story of peptide perception in the root tip even more complex (Stahl and Simon, 2009). In this context, the clade of CR4-related RLKs is widespread in land plants and plays a role in various developmental processes (Nikonorova *et al.*, 2015; Demko *et al.*, 2016). Czyzewicz *et al.* (2016) review what is known about ARABIDOPSIS CR4 (ACR4).

The LRR-RLK XYLEM INTERMIXED WITH PHLOEM 1 (XIP1)/C-TERMINAL ENCODED PEPTIDE RECEPTOR 1 (CEPR1) and CEPR2 were proposed to acts as receptors for CEPs (Tabata *et al.*, 2014). Here, Roberts *et al.* (2016) provide evidence that XIP1 also regulates lateral root initiation and development, and suggest – together with other evidence (Tabata *et al.*, 2014) – that there is a CEP5-XIP1 pair that affects lateral root initiation. Interestingly, it is suggested that in this case CEP5 might inactivate XIP1 and possibly act as an antagonist.

How CRPs are perceived by cells has been less studied. While plant AMPs seem to interact with specific membrane lipids, causing pore formation and subsequent disruption of membranes (Wilmes *et al.*, 2011; Weidmann and Craik, 2016), recent reports provide evidence that CRPs that are involved in controlling stomatal density and patterning, regulating cell expansion, or acting as pollen tube attractants are also perceived by RLKs (Lee *et al.*, 2012; Haruta *et al.*, 2014; Takeuchi and Higashiyama, 2016).

In conclusion, we are gradually gaining insight into peptide–receptor interactions, moving from a handful, largely identified through genetic studies, to several pairs, identified through a wide range of approaches. Very recently, LRR-RLKs have also been identified for RGF/CLEL/GLV peptides (Ou *et al.*, 2016; Shinohara *et al.*, 2016; Song *et al.*, 2016). Given the new tools and approaches available, we expect that the number of known peptide–receptor pairs will quickly increase.

## Future perspectives

A wide range of biochemical and molecular processes are activated downstream of the peptide–receptor kinase interaction (Czyzewicz *et al.*, 2013). However, so far this has been little explored. One interesting example is the RALF–FERONIA (FER) interaction, where the same phosphoproteomics experiment exposed the receptor (FER) and one target (H^+^-ATPase 2, AHA2) (Haruta *et al.*, 2014). In future, more phosphoproteomics-type experiments should reveal additional components of small peptide-triggered signalling cascades. Other downstream factors include transcription factors, such as PLETHORAs (PLTs), which are altered in their expression level and/or abundance (Matsuzaki *et al.*, 2010; Shinohara *et al.*, 2016). In combination with protein–protein interaction studies, this will further lead to a more comprehensive understanding of the signalling complexes. In addition, little is known about the various processing steps and proteins involved, and while some processing enzymes have been identified (Tsiatsiani *et al.*, 2012; Tabata and Sawa, 2014; Wrzaczek *et al.*, 2015), we expect this to be only the tip of the iceberg in generating specificity and activity. Another current shortcoming is the *in planta* visualization of mature peptides, and tools to tag, track and show dynamic *in planta* interaction of small signalling peptides with receptors are required.
